# Sociodemographic disadvantage in the burden of stress and academic performance in medical school: implications for diversity in medicine

**DOI:** 10.1186/s12909-024-05263-y

**Published:** 2024-03-29

**Authors:** Danielle Eames, Shelby Thomas, Kaden Norman, Edward Simanton, Anne Weisman

**Affiliations:** Kirk Kerkorian School of Medicine at UNLV, 625 Shadow Lane, Las Vegas, NV 89106 USA

**Keywords:** Nontraditional, Medical student, Sociodemographic, Stress, Academic performance, Socioeconomic disadvantage, Underrepresented in medicine, First-generation, Older students, Diversity in medicine

## Abstract

**Background:**

Nontraditional students bring to medicine inherent characteristics and perspectives that enrich the learning environment and contribute to expanding diversity in medicine. However, research has shown that these students, by virtue of their sociodemographic backgrounds, face unique challenges in medical education, which ultimately place them at a disadvantage compared to their peers. The purpose of this study is to explore relationships between sociodemographic characteristics, stress, and academic performance, in the context of outcomes that may be undermining efforts to diversify the physician workforce.

**Methods:**

Using a retrospective observational cohort methodology, we examined institutional and USMLE exam performance data in conjunction with Perceived Stress Scale-4 survey results from six cohorts of students at Kirk Kerkorian School of Medicine at UNLV (*n* = 358). Using independent samples t-test, mean stress and academic performance were compared between four sociodemographic groups: first-generation college students, underrepresented in medicine (URM), socioeconomically disadvantaged, and age 30 + at matriculation. Results were considered significant where *P* ≤ .05.

**Results:**

First-generation college students had significantly higher stress at the end of third year clerkships (mean 7.8 vs. 6.8, P* = .03). URM students had significantly lower scores on preclinical exams (mean 81.37 vs. 83.07, P* = .02). The students who were age 30 + at matriculation had significantly lower exam scores on all academic performance measures.

**Conclusion:**

Our results echo historic trends in academic performance for racial and ethnic minority students, and we present recent evidence of academic performance disparities based on age at matriculation. Residency program directors continue to use test scores as a primary metric to screen applicants and thus, poor academic performance has profound consequences on career trajectory. Finally, significantly higher stress in the first-generation students may be evidence of underlying psychological distress. Expanding the sociodemographic diversity among physicians, and by extension, medical students, has long been recognized as fundamental to addressing inequities in healthcare. However, results from our study suggest that aspects of medical education are unfavorable and disadvantageous for first-generation, URM, and older medical students. A deeper understanding of the interplay between sociodemographic characteristics and success in medical school is paramount as we pursue diversity in medicine.

**Supplementary Information:**

The online version contains supplementary material available at 10.1186/s12909-024-05263-y.

## Background

Research has shown that being a medical student is associated with a greater degree of perceived stress, reduced overall wellbeing [1–3], higher rates of depression, anxiety, and burnout [4–7] and that these experiences can negatively impact academic performance [1–3, 8]. The 2022 National College Health Assessment (NCHA) survey, which collects data on factors that students perceive to affect their academic performance, reported that the top three factors cited by graduate and professional students were stress (33%), anxiety (28%), and depression (20%) [8]. Common sources of stress in medical education include assessment-related performance pressure, excessive workload, conflicts in school-life balance and personal relationships, peer relations, health concerns, the learning environment, and administrative failures [6, 7, 9–11]. In addition to these stressors faced by all medical students, nontraditional students^1^ face additional stressors that further compromise health and well-being, detract from academic success, and diminish sense of fulfillment with medical training [1–3, 10, 12–15].

In a study of 69,722 students from more than 100 different U.S. post-secondary institutions, Stevens, et. al. (2018), found that discrimination is a common stressor for racial/ethnic minority undergraduate students, and that these experiences negatively impact their academic performance [16]. These students are also vulnerable to “minority status stress,” which refers to heightened feelings of not belonging that interfere with social integration [17]. Research on older medical students, though sparse, suggests that these students experience a greater overall stress burden due to additional responsibilities outside of medical school and that these responsibilities interfere with studying [18]. Mason et. al. (2018) looked at several indicators of well-being in first-generation medical students. They found significant negative correlations between perceived stress and quality of life across physical, psychological, social, and environmental domains [15]. Studies have shown that financial stress related to excess education debt disproportionately affects racial and ethnic minority, low-income, and first-generation college students [13, 19]. Furthermore, studies show that the accumulation of large amounts of debt during medical school is associated with increased stress [1, 13], poorer academic performance [1], increased risk for burnout [5], and pursuit of higher-paying subspecialties [1, 20].

Nontraditional students bring to medicine inherent characteristics and perspectives that enrich the learning environment [21] and contribute to expanding sociodemographic diversity among physicians [22–24]. However, by virtue of their sociodemographic backgrounds, these students face unique challenges in medical education, which ultimately places them at a disadvantage compared to their peers. The purpose of this study is to explore relationships between sociodemographic characteristics, stress, and academic performance, in the context of outcomes that may be undermining efforts to diversify the physician workforce. Utilizing data collected at Kirk Kerkorian School of Medicine at UNLV, we evaluated stress and academic performance in four categories of nontraditional students with the hypothesis that, when compared to their counterparts, these students would have higher perceived stress, lower academic performance, and possibly both. This study will contribute to the growing body of knowledge on stress and academic performance in medical education for those who approach medical school from a place of sociodemographic disadvantage. To the best of our knowledge, this study is among the first to look at stress, at multiple predetermined points in the curriculum, juxtaposed with academic performance. Notably, we report findings on “older” students, a small but richly diverse subset of nontraditional medical students on which research is considerably lacking.

## Methods

Sociodemographic characteristics, Perceived Stress Scale-4 (PSS) scores, and exam performance data on 358 of 360 students who matriculated to the Kirk Kerkorian School of Medicine as part of the graduating classes of 2021 through 2026 were utilized for the purposes of this retrospective observational cohort study. Due to substantial fluctuations in graduation timelines, 2 students were not included in any of the data analyses. All study participant data were deidentified prior to retrieval and utilized in accordance with existing IRB-approved protocols. The sociodemographic groups, data, and respective analyses are described below.

### Sociodemographic group classification and justification

#### Selection of nontraditional sociodemographic groups

Using data collected at the time of admission, students were sorted into one or more of the following demographic groups: First-Generation College Student (FGCS), Underrepresented in Medicine (URM), Socioeconomically Disadvantaged (SED), and Age 30 years or older (Age 30 +) at matriculation. These groups were chosen based on data published by the Association of American Medical Colleges (AAMC) showing that these groups are currently underrepresented among medical students. Nationally, of the medical school matriculants in 2022, 11.2% were first-generation college students [25], 22.7% were URM [26], 21.5% were socioeconomically disadvantaged [27], and 5.7% were 29 years of age or older [28]. Furthermore, a 2017 analysis of socioeconomic diversity among US medical students found that, in 2017, 24% of matriculants reported parental income in the top 5% (greater than $225,251) of all US households and over half were from households in the top 20% (greater than $121,019), findings that have been consistent for the past 30 years [29]. Based on these data, we considered these categories (e.g., FGCS, URM, SED, and Age 30 +) to be appropriate nontraditional sociodemographic groups to be included in our study.

#### First-Generation College Students: FGCS vs. CGCS

Following the AAMC definition, students whose “most highly educated parent/guardian has up to the equivalent of some college but earned no degree” were included in the “FGCS” group. These students were compared to continuing-generation college students (“CGCS”) [25].

#### Underrepresented in Medicine: URM vs. non-URM

The AAMC defines underrepresented in medicine (URM) as “racial and ethnic populations that are under-represented in the medical profession relative to their numbers in the general population" [30]. This definition is purposefully vague with regards to race and ethnicity to allow for geographic differences and/or temporal changes in population diversity. Students who self-selected “Black/African American”, “Hispanic/Latinx”, and “Native American” (e.g., American Indian, Hawaiian Native, or Alaskan Native) were included in the “URM” group; all other race and ethnic groups were included in the “non-URM group”.

#### Socioeconomic Disadvantage: SED vs. non-SED

Socioeconomic advantage or disadvantage is identified in medical school applicants by combining conventional socioeconomic status (SES) metrics (e.g., annual household income) with additional information regarding parental education and occupation (EO); together, these metrics are known as SES-EO categories, and are stratified into quintiles [29]. Students from households with an annual income below $45,600, or whose parents have “less than a bachelor’s degree, or parents with any degree and a service, clerical, skilled, or unskilled occupation” are classified as SES-EO 1 or 2 and are considered SES-EO disadvantaged. Students from households with annual income above $45,601 or with *at least 1 parent* with “a bachelor’s degree or higher, *and* an executive, managerial, or professional occupation” are grouped into EO-3, EO-4, or EO-5 [29]. Our study group “SED” includes SES-EO 1 and 2 students. Students in SES-EO 3, 4, and 5 categories served as the comparison group “non-SED”.

#### Age at matriculation: age 30 + vs. under 30

To look at differences based on age, we compared students who were 30 years of age or older (“age 30 + ”) to those who were 29 years or younger (“under 30”) at the time of matriculation to medical school. Data from the 2022 AAMC Matriculating Student Questionnaire (MSQ) showed that the vast majority of matriculants were 25 years of age or less (82.9%) [28]. Using this as a reference point, we deemed 30 years of age to be sufficiently different from the average, and thus an appropriate cutoff to represent an “older” medical student.

### Stress data

#### Perceived stress scale-4

As part of continuous internal quality improvement efforts, institutional program evaluation data has been collected from all students beginning in 2017 with the matriculation of the inaugural class at Kirk Kerkorian School of Medicine at UNLV. Part of this evaluation includes assessing student stress using the Short Form Perceived Stress Scale Questionnaire (PSS-4), a widely used tool to quantify perceived general stress [28, 31–33]. The PSS-4 consists of four questions designed to measure perceived stress over the previous month (see Appendix 1 for an outline of the survey). Each item is scored on a scale of 0 (very low stress) to 4 (very high stress), and the cumulative score out of 16 correlates with the degree of perceived stress [31, 32]. PSS-4 surveys are collected at four educational milestones: *(1) prior to matriculation (“pre-matriculation”), (2) at the end of the preclinical phase, (3) at the end of third-year clerkship rotations, and (4) immediately before students participate in the residency matching program (“pre-match”)*. Due to the rolling nature of program evaluation data collection, all students had not participated in data collection at all educational milestones at the time of the study. For example, all 358 students included in the study had completed the pre-matriculation survey, while only 139 students had completed the pre-match survey.

### Academic performance data

#### Institutional NBME exam performance: “preclinical exam average” and "clinical subject exam average”

In the preclinical, organ-systems-based curriculum, student knowledge is assessed using the Customized Assessment Service from the National Board of Medical Examiners (NBME). Each organ-system block varies slightly in length, and, due to evolving curricular structure, each cohort of students have taken a different number of preclinical assessments. For the purposes of comparing academic performance, an average exam score was calculated for each study participant using their scores on all administered preclinical exams. This average exam score for each study participant was then used in the statistical analysis. The results of this analysis are reported as “preclinical exam average”.

Clinical knowledge is assessed during the third year of medical school using Clinical Subject NBME Exams, covering six core specialties (e.g., Internal Medicine, Family Medicine, Pediatrics, Obstetrics and Gynecology, Surgery, and Psychiatry). As described for the preclinical exams, the average exam score calculated for each study participant was used in the statistical analysis. The results of this analysis are reported as “clinical subject exam average”.

#### USMLE performance: “Step 1” and “Step 2 CK”

Three-digit numeric scores on the Step 1 and Step 2 CK United States Medical Licensing Examinations (USMLE) for each study participant were used in the statistical analysis. Students sit for Step 1 after the preclinical phase and must pass the exam before being promoted to the clinical phase of the curriculum. Students may sit for Step 2 CK at any time after Step 1. Therefore, at the time of this study, some students may have taken none, one, or both USMLE exams. Additionally, we did not include students who took the USMLE Step 1 after January 26, 2022, when the exam moved to Pass/Fail score reporting. Results of this statistical analysis are reported as “Step 1 average” and “Step 2 CK average”.

### Statistical analysis

Using these preexisting data sets, independent samples t-tests were calculated with Statistical Package for Social Sciences (SPSS) (version 27) software to assess for differences in PSS-4 scores and academic performance between *FGCS vs. CGCS; URM vs. non-URM; SED vs. non-SED;* and *Age 30* + *vs. Under 30.* Results were considered significant (indicated as P*) for two-sided *P* values where *P* ≤ 0.05. All *P* values are reported with equal variances assumed unless otherwise noted.

## Results

Of the 358 students included in the study, nearly all of them (97.2%) fall into at least one of our nontraditional sociodemographic groups (Table 1).

**Table 1:** Sociodemographic data

Characteristic	N (%)
First-generation college student (FGCS)	103 (28.8%)
Underrepresented in medicine (URM)	74 (20.7%)
Socioeconomic disadvantaged (SED)	137 (38.3%)
Age 30 or older at matriculation (Age 30 +)	34 (9.5%)

Number and percentage of study participants in each of the four sociodemographic groups

*Abbreviations*: *N* Number of study participants

### FGCS vs. continuing-generation college students (CGCS)

**Fig. 1:**
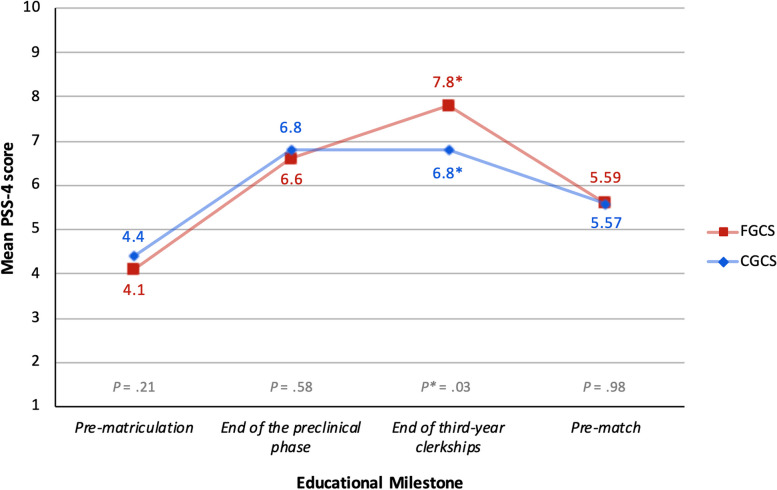
Results of mean PSS-4 score comparison between FGCS vs. CGCS. While actual scores can range from 0 to 16, the y-axis has been amended to a range of 1 to 10 in order to visually present the data. *Abbreviations*: *FGCS* first-generation college student, *CGCS* continuing-generation college student, *PSS-4* Perceived Stress Scale-4

Compared to their CGCS peers, we found that FGCS had lower stress at the first two educational milestones (e.g., pre-matriculation and the end of the preclinical phase), roughly the same stress at the final educational milestone (e.g., as students approached the residency match), but, between these points, at the end of third-year clerkships, stress among the FGCS was significantly higher than that of their CGCS counterparts (mean 7.8 vs. 6.8; 95% CI [0.09 to 1.98], P* = 0.03) (Fig. 1; see also Supplementary Table 1, Appendix 2). While the FGCS and CGCS performed roughly the same on the preclinical institutional exams, the FGCS had lower average scores on all other academic performance measures. However, none of these differences met statistical significance (Table 2).

**Table 2:** Academic performance: first-generation vs. continuing-generation college students

Academic Performance Measure	Student status	N	Mean (SD)	*P* value	95% CI
Preclinical exam average (%)	FGCS	85	82.54 (4.60)	.70	-1.56 to 1.06
	CGCS	210	82.80 (5.44)		
Clinical subject exam average (%)	FGCS	50	77.67 (5.57)	.46	-2.60 to 1.19
	CGCS	124	78.38 (5.79)		
Step 1 average (3-digit score)	FGCS	50	227.3 (14.6)	.12	-9.29 to 1.07
	CGCS	133	231.4 (16.3)		
Step 2 CK average (3-digit score)	FGCS	50	244.1 (14.4)	.20	-7.48 to 1.68
	CGCS	124	247.2 (14.4)		

Results of mean academic performance between FGCS vs. CGCS

*Abbreviations*: *FGCS* First-generation college student, *CGCS* Continuing-generation college student, *N* Number of study participants, *SD* Standard deviation, *CI* Confidence interval

### Underrepresented in Medicine (URM) vs. non-URM

**Fig. 2:**
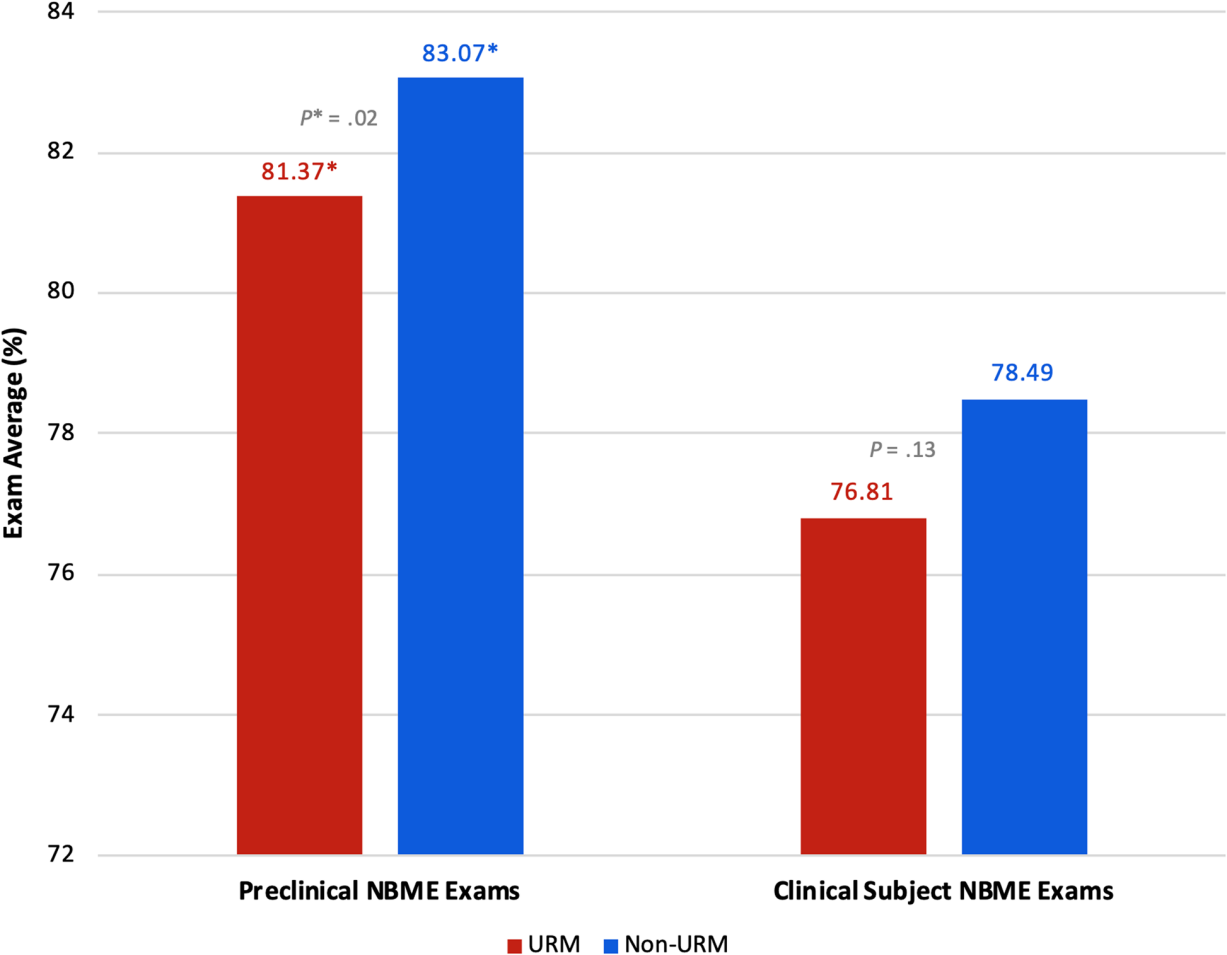
Results of institutional preclinical and clinical subject NBME exam performance between URM vs. non-URM students. While actual scores can range from 0 to 100, the y-axis has been amended to a range of 72 to 84 in order to visually present the data. *Abbreviations*: *URM* underrepresented in medicine, *NBME* National Board of Medical Examiners

**Fig. 3:**
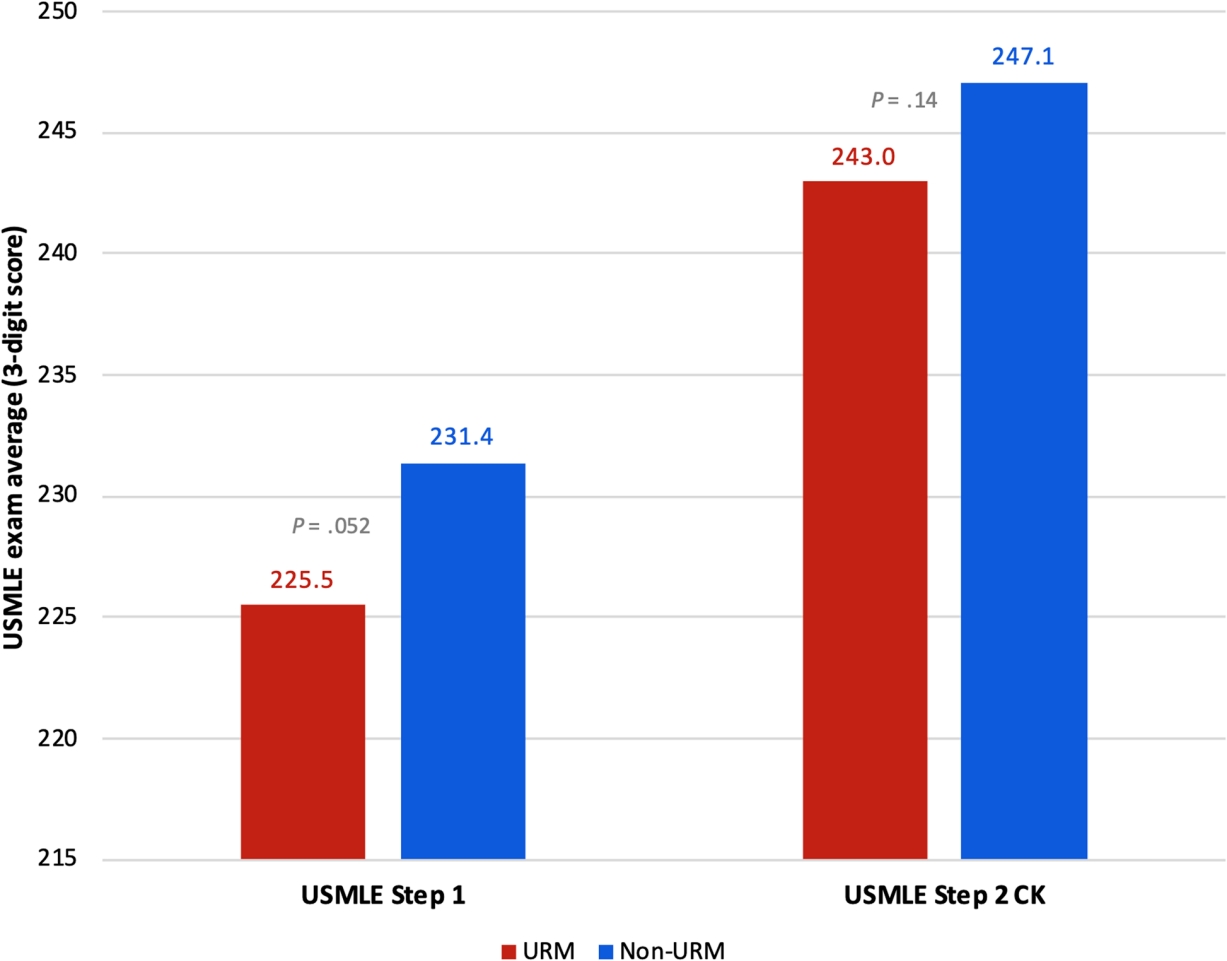
Results of USMLE performance between URM vs. non-URM students. While actual scores can range from 0 to 300, the y-axis has been amended to a range of 215 to 250 in order to visually present the data. *Abbreviations*: *URM* underrepresented in medicine, *USMLE* United States Medical Licensing Examination

The comparison of stress between URM and non-URM students showed that URM students had lower stress at each educational milestone until the last PSS-4 survey collection, immediately before students participate in the residency match (“pre-match”). However, none of the differences in stress between URM and non-URM students met statistical significance (Table 3). When we compared academic performance, we found that URM students had lower average exam scores on institutional and USMLE exams; however, the only statistically significant difference was performance on the preclinical NBME exams (mean 81.37 vs. 83.07; 95% CI [-3.17 to -0.23], P* = 0.02) (Figs. 2 and 3; see also Supplementary Table 2, Appendix 2).

**Table 3:** Stress: URM vs. non-URM

Educational Milestone	Student status	N	Mean (SD)	*P* value	95% CI
Pre-Matriculation	URM	74	3.9 (2.6)	.13	-0.94 to 0.21
	non-URM	284	4.4 (2.5)		
End of preclinical phase	URM	60	6.8 (3.2)	.83	-0.98 to 0.55
	non-URM	234	6.7 (3.0)		
End of third year clerkships	URM	44	6.9 (2.7)	.69	0.09 to 1.98
	non-URM	187	7.1 (3.4)		
Pre-Match	URM	22	5.8 (3.6)	.77^a^	-1.06 to 1.08
	non-URM	117	5.5 (2.8)		

Results of mean PSS-4 score comparison between URM vs. non-URM students

*Abbreviations*: *URM* Underrepresented in medicine, *N* Number of study participants, *SD* Standard deviation, *CI* Confidence interval

^a^equal variances NOT assumed

### Socioeconomic Disadvantage (SED) vs. non-SED

Socioeconomically disadvantaged (SED) students had higher stress at the first three educational milestones (e.g., pre-matriculation, the end of the preclinical phase, and the end of third-year clerkships) (Table 4), and lower average performance on the clinical subject and USMLE Step 1 exams (Table 5) compared to their non-SED counterparts. However, these differences were not statistically significant.

**Table 4:** Stress: SED vs. non-SED

Educational Milestone	Student status	N	Mean (SD)	*P* value	95% CI
Pre-Matriculation	SED	137	4.6 (2.6)	.14	-0.13 to 0.94
	non-SED	221	4.2 (2.4)		
End of preclinical phase	SED	111	7.0 (2.9)	.24	-0.28 to 1.14
	non-SED	183	6.6 (3.0)		
End of third year clerkships	SED	80	7.4 (3.4)	.25	-0.37 to 1.40
	non-SED	151	6.9 (3.1)		
Pre-Match	SED	52	5.5 (3.1)	.72	-1.19 to 0.83
	non-SED	87	5.6 (2.8)		

Results of mean PSS-4 score comparison between SED vs. non-SED students

*Abbreviations*: *SED* Socioeconomic disadvantage, *N* Number of study participants, *SD* Standard deviation, *CI* Confidence interval

**Table 5:** Academic performance: SED vs. non-SED

Academic Performance Measure	Student status	N	Mean (SD)	*P* value	95% CI
Preclinical exam average (%)	SED	111	82.62 (5.12)	.80	-1.39 to 1.07
	non-SED	184	82.78 (5.27)		
Clinical subject exam average (%)	SED	69	77.88 (5.93)	.57	-2.26 to 1.25
	non-SED	105	78.38 (5.60)		
Step 1 average (3-digit score)	SED	71	228.5 (15.7)	.21	-7.78 to 1.17
	non-SED	112	231.5 (15.9)		
Step 2 CK average (3-digit score)	SED	69	246.7 (14.0)	.79	-3.82 to 5.02
	non-SED	105	246.1 (14.8)		

Results of mean academic performance comparison between SED vs. non-SED students

*Abbreviations*: *SED* Socioeconomic disadvantage, *N* Number of study participants, *SD* Standard deviation, *CI* Confidence interval

### Age 30 + at matriculation vs. under 30

**Fig. 4:**
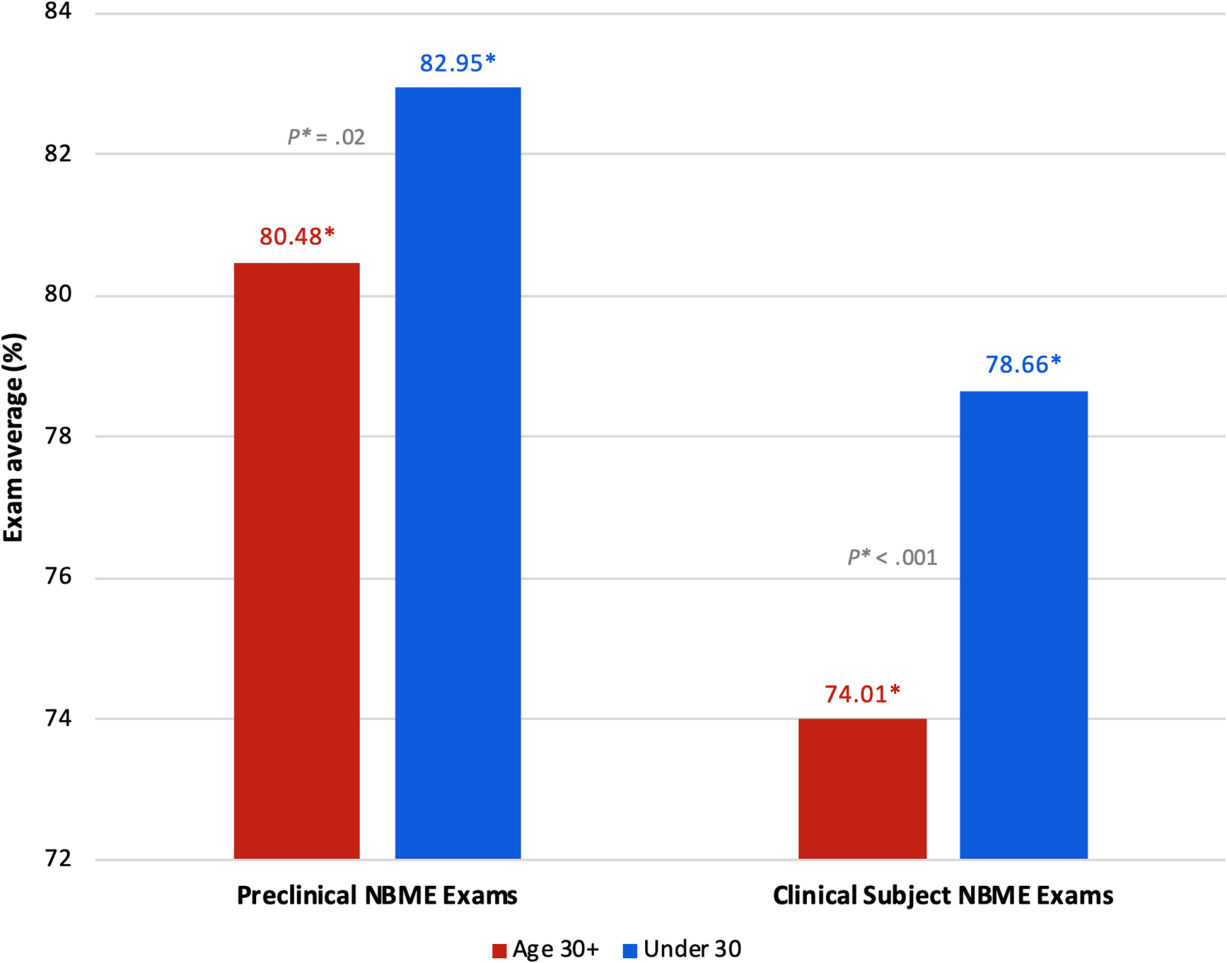
Results of institutional NBME exam performance between students age 30 + vs. under 30 at matriculation. While actual scores can range from 0 to 100, the y-axis has been amended to a range of 72 to 84 in order to visually present the data. *Abbreviations*: *NBME* National Board of Medical Examiners

**Fig. 5:**
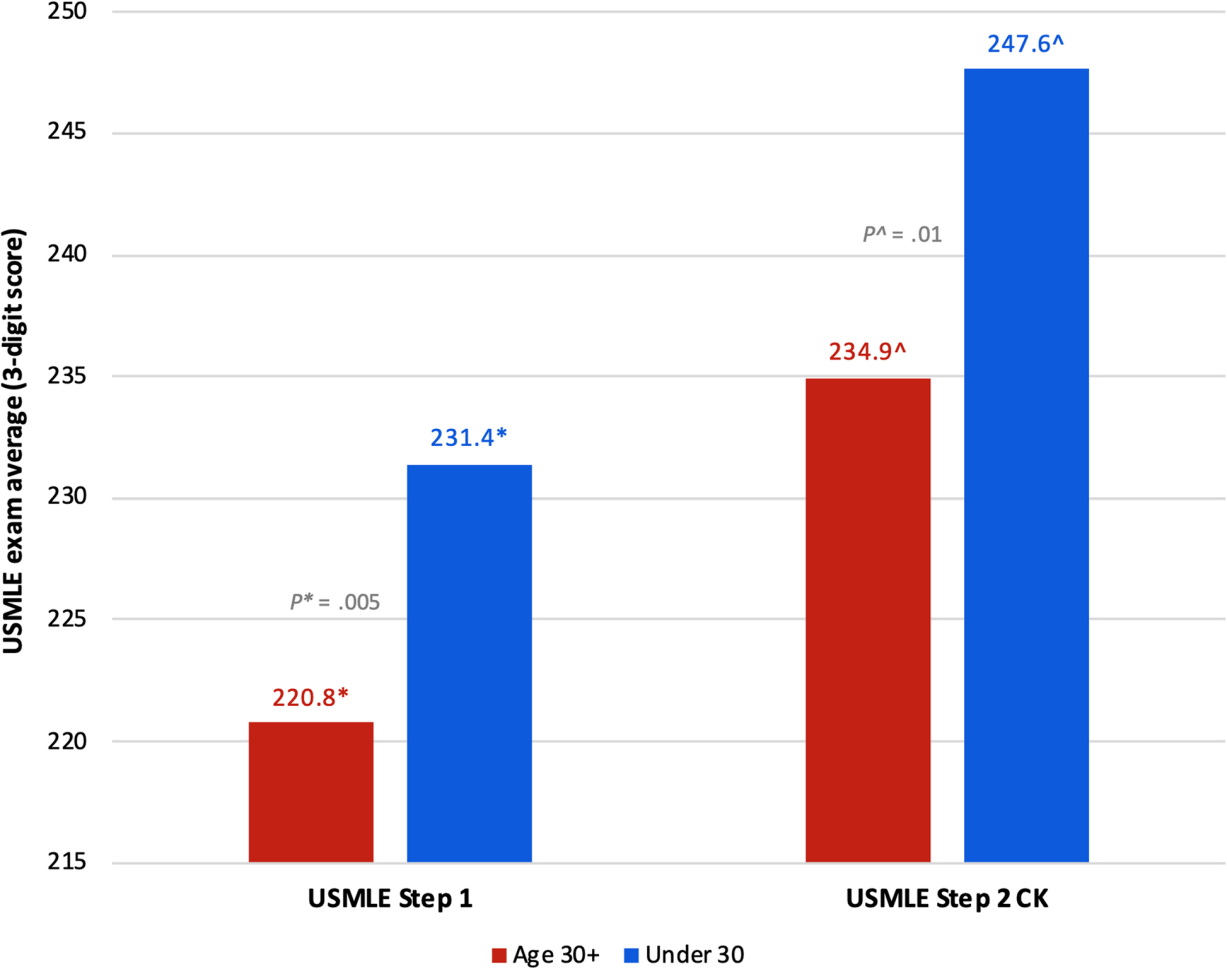
Results of USMLE performance between students age 30 + vs. under 30 at matriculation. While actual scores can range from 0 to 300, the y-axis has been amended to a range of 215 to 250 in order to visually present the data. *Abbreviations*: *USMLE* United States Medical Licensing Examination ^equal variances NOT assumed

The comparison of stress between the Age 30 + and Under 30 students revealed lower stress among the Age 30 + cohort at the first three educational milestones, and higher stress only before the residency match (Table 6); however, none of these differences were statistically significant. When we compared academic performance between these two groups, the Age 30 + students had significantly lower average exam scores across all academic performance measures (Figs. 4 and 5; see also Supplementary Table 3, Appendix 2). Results of institutional exam performance are shown in Fig. 4, with students who were Age 30 + at matriculation scoring significantly lower on the preclinical exams (mean 80.48 vs. 82.95; 95% CI [-4.52 to -0.42]; P* = 0.02) and the clinical subject exams (mean 74.01 vs. 78.66; 95% CI [-7.39 to -1.92]; P* < 0.001). When we looked at USMLE performance (Fig. 5), we again found that students who were Age 30 + at matriculation scored significantly lower on both Step 1 (mean 220.8 vs. 231.4; 95% CI [-18.07 to -3.17]; P* = 0.005) and Step 2 CK (mean 234.9^ vs. 247.6^; 95% CI [-22.11 to -3.30]; *P^* = 0.01, equal variance NOT assumed). Of note, the statistical analysis of USMLE Step 2 CK performance in this group violated the assumption of equal variances. Nevertheless, the results are included in the figure for transparency and visual continuity.

**Table 6:** Stress: Age 30 + vs. Under 30 at matriculation

Educational Milestone	Student status	N	Mean (SD)	*P* value	95% CI
Pre-Matriculation	Age 30 +	34	3.9 (2.7)	.33	-1.33 to 0.45
	Under 30	324	4.4 (2.5)		
End of preclinical phase	Age 30 +	27	6.6 (3.5)	.81	-1.34 to 1.05
	Under 30	267	6.7 (3.0)		
End of third year clerkships	Age 30 +	25	6.5 (3.1)	.37	-1.97 to 0.74
	Under 30	206	7.1 (3.3)		
Pre-Match	Age 30 +	13	6.2 (3.4)	.45	-1.03 to 2.31
	Under 30	126	5.5 (2.8)		

Results of mean PSS-4 score comparison between students age 30 + vs. under 30 at matriculation

*Abbreviations*: *N* Number of study participants, *SD* Standard deviation, *CI* Confidence interval

## Discussion

### Academic performance

URM students scored lower, on average, than their non-URM peers on all academic performance measures, with statistically significant lower average performance on the preclinical exams (Figs. 2 and 3; see also Supplementary Table 2, Appendix 2). Although these results were only partially statistically significant, this trend in academic performance among URM students is important because it echoes historic trends in assessment performance disparities impacting racial and ethnic minority students [34–38]. Research on the achievement gap in medical education has highlighted how structural inequities in education, stemming from poorly funded K-12 schools that primarily serve low-income and minority children, lead to disparities in performance on standardized exams, including the Medical College Admission Test (MCAT) and United States Medical Licensing Examinations (USMLE) [34–36]. The most recent AAMC report on medical school applicants and matriculants, which included data from the 2022–2023 application cycle, showed that URM students (Black/African American, Hispanic/Latino/Spanish, and American Indian/Alaskan Native/Native Hawaiian/Pacific Islander) had lower average GPAs and MCAT scores than non-URM students [39]. A 2019 study led by a team at the National Board of Medical Examiners (NBME), found that Hispanic and Black students were significantly more likely to score lower on any of the Step exams compared to White students, and that nearly half of all students who initially fail Step 1 are racial/ethnic minority students [37].

Students who were 30 years of age or older at matriculation had significantly lower average exam scores across all academic performance measures compared to their younger counterparts (Figs. 4 and 5; see also Supplementary Table 3, Appendix 2). These findings indicate that there is a need that is not being met for these students. Previous research, though considerably outdated, has suggested that older medical students may have different learning strategies than their younger counterparts. In a 1998 study at McGill University, Feil et. al. found that older medical students approached learning more abstractly, with concern for the thought processes involved in basic and clinical science, while younger students were more inclined to study by memorizing facts for a test for the sake of getting good grades [40]. In 2000, Kick et. al. reported that older students perceived medical school to be more intrusive upon their deeply developed personal lives, and that responsibilities at home made it difficult to study [18]. Given the paucity of recent research on the experiences of older medical students, our findings contribute immensely to the literature. Older medical students bring valuable life experience to patient care and further research to identify factors that may be undermining academic performance among these students is necessary.

### Stress

Surprisingly, the only comparison of stress that showed statistically significant higher stress among any of our study groups was between first-generation and continuing-generation college students at the end of third-year clerkships (Fig. 1; see also Supplementary Table 1, Appendix 2). We posit that, because we found evidence of significantly higher perceived stress among *only* first-generation college students and *specifically* at the end of third year clerkships, a time when FGCS are exposed to a variety of unfamiliar clinical settings and a highly competitive learning environment, this unique finding may be related to imposter phenomenon.

Imposter phenomenon is characterized by an overwhelming belief that one does not belong in a certain setting despite evidence to the contrary and fear about being discovered as a “fraud” [41]. People experiencing imposter phenomenon have chronic self-doubt and are unable to internalize personal achievements [41]. Levant, et. al. (2020) found significant correlations between stress (measured with the 10-item perceived stress scale [PSS-10]) and imposter feelings (measured with the Clance Impostor Phenomenon Scale), and that PSS-10 scores were 28–31% higher in those experiencing imposter phenomenon [42]. Studies on FGCS at the undergraduate level have shown that these students more frequently report difficulty fitting into campus culture and often doubt their abilities to succeed, feelings that are directly related to imposter syndrome [43, 44]. Canning, et. al. (2019) looked at associations between peer competition, generational status, and imposter feelings in students taking Science, Technology, Engineering, and Mathematics (STEM) undergraduate courses [45]. They found that FGCS were significantly more likely to experience imposter feelings in settings where perceived competition was increased (e.g., STEM courses) [45]. Although further investigation is needed to make a definitive conclusion about the source of the increased stress in this study group, our results parallel that of previous research on the experience of imposter phenomenon among first-generation college students. This theory is further supported by the *absence* of significant reductions in exam performance among the FGCS in our study, as this provides evidence that these students are not academically inferior to their peers.

### Negative findings

In none of the study groups did we find both higher stress *and* lower academic performance. In fact, our results show that URM and Age 30 + students arrived at medical school with the lowest reported stress of any group (Tables 3 and 6, respectively). Based on socioeconomic disadvantage (SED), no significant differences in stress *or* academic performance were found. Our lack of any significant differences among SED students compared to their non-SED counterparts, particularly with regards to stress, was surprising, as the association of debt and stress is well-established and existing literature has shown increased stress in these students [1, 13]. Given the sociodemographic diversity of our study participants (Table 1), it would be reasonable to infer that a culture of inclusion and acceptance exists among the students, which may be protective against stress and its sequela.

Additionally, these findings may reflect individual differences in stress appraisal and resilience. Research on stress theory holds that the effects of stress depend, at least in part, on whether the stress is perceived as enhancing or debilitating [46]. For those who perceive stress as enhancing, it can improve performance and enhance motivation to overcome a challenge [46]. Perhaps the FGCS in our study, while they have significantly higher stress than CGCS at the end of third-year clerkships, may be less inclined to appraise stress as a negative factor, and thus academic performance was not impacted. The older students significantly under-performed on all academic measures (Figs. 4 and 5) yet report some of the lowest stress levels of anyone until they approach the residency match (Table 6). Studies on older medical students indicate that they are more likely to hold an internal locus of control, demonstrate greater critical thinking abilities, and have an increased propensity for self-reflection [47, 48]. Having led full lives, with prior careers and other life experience, it is possible the older students approach the burden of medical school differently and may be more accepting of their personal limitations. Moreover, by virtue of their nontraditional sociodemographic backgrounds, these students may possess greater resilience. If our nontraditional students have lived experiences overcoming greater adversity, then they may be less inclined to perceive, or report, increases in stress.

### Limitations

Our study has several limitations. Our unusually diverse but small study population involving students from a single institution may limit the generalizability of our results. It is likely that our small sample size contributes to many of our comparisons being found statistically non-significant. Our statistical analysis does not account for students who fall into multiple sociodemographic groups. The collection of program evaluation data by Kirk Kerkorian School of Medicine necessitates that the surveys be a required component of the curriculum. Because of this, it is possible that our results were confounded by individual differences in attention and reflection on the survey questions. While the PSS-4 was designed to be better suited for settings in which respondents may not have the time or desire to complete the longer versions of the PSS (e.g., the 10-item and 14-item questionnaires) [31], and indeed this was the rationale for using the Short Form PSS, it is possible for students to simply click though the survey because it is required to do so without responding thoughtfully to the survey questions. If this is the case, then the survey results would not reflect what we are trying to measure. Lastly, the statistical analysis of differences in USMLE Step 2 CK performance in the “age 30 + ” group violated the assumption of equal variances which remains unexplained by our data set.

### Implications

Expanding the sociodemographic diversity among physicians, and by extension, medical students, has long been recognized as fundamental to addressing healthcare inequities in the US [49]. In 2009, the Liaison Committee on Medical Education (LCME) began implementing accreditation standards regarding the benefits of diversity, which now also include policies on anti-discrimination, cultural competency, and addressing disparities in social determinants of health [50–52]. Today, considerable effort is put towards increasing the matriculation of students from nontraditional sociodemographic backgrounds. Medical schools have universally adopted holistic admissions policies that recognize socioeconomic status, demographic characteristics, and life experiences of applicants to encourage the matriculation of nontraditional students [24, 53–55]. Pipeline programs have been implemented in some areas to recruit students to medicine from community colleges, where many nontraditional students begin their post-secondary education [22–24, 36, 56, 57]. Despite these efforts, however, disparities in the availability and quality of healthcare resources, burden of cost, health insurance coverage, patient outcomes, general health status, and overall life expectancy continue to exist for racial/ethnic minority, low-income, and inner city, and rural communities [58].

The purpose of this study was to explore relationships between sociodemographic characteristics, stress, and academic performance, in the context of outcomes that may be undermining efforts to diversify the physician workforce. Nontraditional students bring to medicine inherent characteristics and perspectives that enrich the learning environment [21], and contribute to expanding access to culturally competent, linguistically appropriate healthcare services for underserved communities in the US [22–24]. With a projected shortage of up to 124,000 physicians by 2034 [59], and the significant lag time between embarking on post-secondary education and independent practice, increasing the diversity among medical students is more urgent than ever. Results from our study suggest that aspects of medical education are unfavorable and disadvantageous for first-generation, URM and older medical students. Residency program directors continue to use USMLE test scores as a primary metric to screen applicants [60]. Therefore, poor performance on these exams has profound consequences on career trajectory which, in turn, may be impeding progress towards increasing diversity in medicine. The increased stress in first-generation students at the end of third year clerkships may be indicative of underlying psychological distress.

A deeper understanding of the interplay between sociodemographic characteristics and success in medical school, both psychosocially and academically, is paramount if we are to achieve diversity in medicine that matches that of the population and, ultimately, health equity. Our study looked at stress, which is just one possible cause for nontraditional students to be unsuccessful in medical school, and we looked at academic performance, which is just one possible measure of success or failure in medical school. The PSS-4 captures general stress at moments in time, but it tells us nothing about the quality of stress. Further investigation employing qualitative methods could elucidate sources of stress and factors undermining academic performance for nontraditional students. While USMLE exam performance continues to be a critical component of a student’s competitiveness for residency programs, it is closely followed by narrative evaluations [60]. Moreover, there is evidence that narrative evaluations, which are inherently subjective, are prone to both implicit and explicit bias, thereby adding another element of disadvantage for certain groups of nontraditional students [35, 61, 62]. To fully understand the experience of nontraditional medical students, exploring the effect of these evaluations, both internally at our institution and broadly across the US, is necessary. It is incumbent upon medical educators to support their students in meaningful ways and to promote the success of nontraditional students. This may be through individualized support, both academically and personally, flexible curricula to meet the needs of a diverse student body, or through system-level changes that lead to learning environments more favorable to nontraditional students.

## Conclusion

Results from our study echo historic trends in academic performance for racial and ethnic minority students, with our URM students scoring significantly lower on the standardized preclinical NBME exams than their non-URM peers. Additionally, we present recent evidence of academic performance disparities based on age at matriculation, with our Age 30 + study group significantly underperforming on both institutional exams and national licensing exams. Our results also show significantly higher stress at the end of the third year of medical school for first-generation students, which may be evidence of imposter phenomenon, though further investigation is needed to make that conclusion definitively. Contrary to the literature, however, we did not find any differences based on socioeconomic disadvantage; we attribute this inconsistency to limitations imparted on our study by the nature of the study design itself.

### Supplementary Information

**Supplementary Material 1.**

**Supplementary Material 2.**

## Data Availability

Data available upon request. Please send data requests to Edward Simanton PhD, edward.simanton@unlv.edu.

## Abbreviations

USMLE: United States Medical Licensing Examination
URM: Underrepresented in medicine
NCHA: National College Health Assessment
PSS: Perceived Stress Scale
IRB: Internal review board
FGCS: First-generation college student
URM: Underrepresented in medicine
SED: Socioeconomically Disadvantaged
AAMC: Association of American Medical Colleges
US: United States
CGCS: Continuing-generation college students
SES: Socioeconomic status
EO: Education-Occupation
SES-EO: Socioeconomic Status-Education Occupation
MSQ: Matriculating Student Questionnaire
PSS-4: Perceived Stress Scale-4 (short form)
NBME: National Board of Medical Examiners
USMLE: United States Medical Licensing Examination
CK: Clinical Knowledge
SPSS: Statistical Package for Social Sciences
FGCS: First-generation college student
CGCS: Continuing-generation college student
URM: Underrepresented in medicine
PSS-4: Perceived Stress Scale-4 (short form)
SED: Socioeconomic disadvantage
USMLE: United States Medical Licensing Examination
Step 2 CK: Step 2 Clinical Knowledge
CI: Confidence interval
URM: Underrepresented in medicine
MCAT: Medical College Admission Test
USMLE: United States Medical Licensing Examination
AAMC: Association of American Medical Colleges
GPA: Grade-point average
NBME: National Board of Medical Examiners
FGCS: First-generation college student
PSS-10: Perceived Stress Scale-10 (10-item questionnaire)
STEM: Science, technology, engineering, and mathematics
SED: Socioeconomic disadvantage
CGCS: Continuing-generation college students
PSS-4: Perceived Stress Scale-4 (short form)
LCME: Liaison Committee on Medical Education
US: United States
URM: Underrepresented in medicine
NBME: National Board of Medical Examiners

## Footnotes

1 Nontraditional student refers to students who are considered first-generation college students, underrepresented in medicine minority, socioeconomically disadvantaged, or took any path to medical school other than completing high school, then completing a bachelor’s degree 4 years later, then immediately matriculating to medical school.
