# Supplementary material for: Sociodemographic disadvantage in the burden of stress and academic performance in medical school: implications for diversity in medicine
Source: BMC Med Educ. 2024 Mar 29;24:348. doi: 10.1186/s12909-024-05263-y (PMC10981295; doi:10.1186/s12909-024-05263-y)
Supplement: Supplementary file 1 — Supplementary Material 1. [file 12909_2024_5263_MOESM1_ESM.pdf]

APPENDIX 1 – Perceived Stress Scale-4 questionnaire

Instructions: The questions in this scale ask you about your feelings and thoughts **during the last month**. In each case, please indicate your response by selecting the option representing *how often* you felt or thought a certain way.

In the last month, how often have you:

- felt that you were unable to control the important things in your life?
  - Never / Almost never / Sometimes / Fairly often / Very often
- felt confident about your ability to handle your personal problems?
  - Never / Almost never / Sometimes / Fairly often / Very often
- felt that things were going your way?
  - Never / Almost never / Sometimes / Fairly often / Very often
- felt difficulties were piling up so high that you could not overcome them?
  - Never / Almost never / Sometimes / Fairly often / Very often

---

Scoring Instructions: Total score is determined by adding together the scores of each of the four items. Questions 2 and 3 are reverse coded.

- Questions 1 and 4:
  - Never = 0
  - Almost never = 1
  - Sometimes = 2
  - Fairly often = 3
  - Very often = 4
- Questions 2 and 3:
  - Never = 4
  - Almost never = 3
  - Sometimes = 2
  - Fairly often = 1
  - Very often = 0

REFERENCES

1. Cohen S, Kamarck T, Mermelstein R: **A Global Measure of Perceived Stress.** Journal of health and social behavior 1983, 24(4):385-396. https://www.jstor.org/stable/2136404 DOI:10.2307/2136404
2. Warttig SL, Forshaw MJ, South J, White AK: **New, normative, English-sample data for the Short Form Perceived Stress Scale (PSS-4).** J HEALTH PSYCHOL 2013, 18(12):1617-1628. https://10.1177/1359105313508346 DOI:10.1177/1359105313508346
