# Supplementary material for: Sociodemographic disadvantage in the burden of stress and academic performance in medical school: implications for diversity in medicine
Source: BMC Med Educ. 2024 Mar 29;24:348. doi: 10.1186/s12909-024-05263-y (PMC10981295; doi:10.1186/s12909-024-05263-y)
Supplement: Supplementary file 2 — Supplementary Material 2. [file 12909_2024_5263_MOESM2_ESM.pdf]

APPENDIX 2 – Supplementary data tables

Supplementary Table 1 Stress: first-generation vs. continuing-generation college students

Educational Milestone	Student status	N	Mean (SD)	P value	95% CI
Pre-Matriculation	FGCS	103	4.1 (2.6)	.21	-0.94 to 0.21
	CGCS	255	4.4 (2.4)		
End of preclinical phase	FGCS	84	6.6 (3.0)	.58	-0.98 to 0.55
	CGCS	210	6.8 (3.0)		
End of third-year clerkships	FGCS	61	7.8 (3.1)	.03	0.09 to 1.98
	CGCS	170	6.8 (3.3)		
Pre-Match	FGCS	41	5.59 (2.91)	.98	-1.06 to 1.08
	CGCS	98	5.57 (2.91)		

Results of mean PSS-4 score comparison between FGCS vs. CGCS
***Abbreviations*: *FGCS* first-generation college student, *CGCS* continuing-generation college student, *N* number of study participants, *SD* standard deviation, *CI* confidence interval**

Supplementary Table 2 Academic performance: URM vs. non-URM

Academic Performance Measure	Student status	N	Mean (SD)	P value	95% CI
Preclinical exam average (%)	URM	60	81.37 (4.67)	.02	-3.17 to -0.23
	non-URM	235	83.07 (5.29)		
Clinical subject exam average (%)	URM	32	76.81 (5.98)	.13	-3.88 to 0.52
	non-URM	142	78.49 (5.64)		
Step 1 average (3-digit score)	URM	34	225.5 (15.7)	.052	-11.78 to 0.04
	non-URM	149	231.4 (15.8)		
Step 2 CK average (3-digit score)	URM	32	243.0 (15.2)	.14	-9.67 to 1.43
	non-URM	142	247.1 (14.2)		

Results of mean academic performance between URM vs. non-URM students
***Abbreviations*: *URM* underrepresented in medicine, *N* number of study participants, *SD* standard deviation, *CI* confidence interval**

Supplementary Table 3 Academic performance: Age 30+ vs. Under 30

Academic Performance Measure	Student status	N	Mean (SD)	P value	95% CI
Preclinical exam average (%)	Age 30+	27	80.48 (5.63)	.02	-4.52 to -0.42
	Under 30	268	82.95 (5.12)		
Clinical subject exam average (%)	Age 30+	18	74.01 (6.29)	< .001	-7.39 to -1.92
	Under 30	156	78.66 (5.47)		
Step 1 average (3-digit score)	Age 30+	19	220.8 (15.5)	.005	-18.07 to -3.17
	Under 30	164	231.4 (15.6)		
Step 2 CK average (3-digit score)	Age 30+	18	234.9 (18.5)	.01[a]	-22.11 to -3.30
	Under 30	156	247.6 (13.3)		

Results of mean academic performance between students age 30+ vs. under 30 at matriculation
***Abbreviations*: *N* number of study participants, *SD* standard deviation, *CI* confidence interval**
[a] equal variances NOT assumed
